# Prevention of congenital syphilis: The current state of legislation and public health guidelines

**DOI:** 10.1002/pmf2.70110

**Published:** 2025-09-12

**Authors:** Rose Darcy, Laurie Ayala, Maura Quinlan, Danucha Brikshavana, Anne Statton, Irina Tabidze, Helen Cejtin, Jennifer Jao, Sarah Sutton, Nigel Madden, Mariana Espinal, Lynn M. Yee

**Affiliations:** ^1^ Division of Maternal‐Fetal Medicine, Department of Obstetrics and Gynecology Northwestern University Feinberg School of Medicine Chicago Illinois USA; ^2^ Illinois Perinatal Syphilis Warmline and Illinois Perinatal HIV Hotline Chicago Illinois USA; ^3^ Illinois Department of Public Health Springfield Illinois USA; ^4^ Mother and Child Alliance Chicago Illinois USA; ^5^ Chicago Department of Public Health Chicago Illinois USA; ^6^ Department of Obstetrics and Gynecology John H. Stroger, Jr. Hospital of Cook County Chicago Illinois USA; ^7^ Division of Infectious Diseases, Department of Pediatrics Ann & Robert H. Lurie Children's Hospital of Chicago and Northwestern University Feinberg School of Medicine Chicago Illinois USA; ^8^ Division of Infectious Diseases, Department of Medicine Northwestern University Feinberg School of Medicine Chicago Illinois USA; ^9^ Department of Medicine Northwestern University Feinberg School of Medicine Chicago Illinois USA; ^10^ Division of Maternal‐Fetal Medicine, Department of Obstetrics and Gynecology Beth Israel Deaconess Medical Center Boston Massachusetts USA

**Keywords:** congenital syphilis, perinatal syphilis, pregnancy, public health, syphilis testing

## Abstract

Syphilis infection among pregnant people and congenital syphilis rates have risen considerably in the United States, leading to adverse maternal and neonatal health outcomes. Most cases of congenital syphilis are preventable, but diagnosis and treatment are complex and frequently complicated by stigma and limited access to care. Screening guidelines for perinatal syphilis in the United States vary by state, government agency, and professional organization. The Centers for Disease Control and Prevention (CDC) recommend universal screening of pregnant patients in the first trimester and repeat screening for high‐risk patients at 28 weeks’ gestation and at delivery, while the American College of Obstetricians and Gynecologists (ACOG) newly recommends universal screening at all three time points. Legislation meets CDC and ACOG guidelines in only 46% and 17% of states, respectively. In response to the congenital syphilis crisis, 25 states’ health departments have issued advisories recommending more frequent screening. Despite testing state‐level guidelines, opt‐out screening and legislation do not benefit all patients equally; different rates of syphilis by race, ethnicity, and socioeconomic status highlight the need for additional resources and public health support. Moreover, perinatal syphilis remains challenging due to fragmented health care in the United States. Future directions to optimize public health support for congenital syphilis prevention include developing public health–clinical–academic partnerships that address some of these gaps by providing expert assistance to clinical teams, centralizing reporting and data availability, and supporting patients via novel models of care.

## 1 INTRODUCTION

Syphilis is a contagious sexually transmitted infection (STI) that is caused by infection with the spirochete *Treponema pallidum*. In recent years, rates of primary and secondary syphilis in people of reproductive age have increased considerably, leading to a dramatic 755% rise in rates of congenital syphilis (CS) from 2012 to 2021 [1, 2]. The Centers for Disease Control and Prevention (CDC) estimated a 219% increase in CS cases from 2017 to 2021, from 24.4 to 77.9 cases per 100,000 live births annually [1]. Increases in CS appear to be slowing in some areas—with a 3% increase over 2022 nationally, compared to 30% annual increases in prior years [2, 3].

The consequences of unrecognized syphilis during pregnancy are significant, including adverse short‐ and long‐term maternal and neonatal health outcomes and significant health care spending [4, 5]. Perinatal transmission of syphilis costs an estimated $3.6 million in disability‐adjusted life‐years and $309 million in medical costs globally [5, 6]. CS causes widespread inflammation that can damage fetal organ systems, contributing to pregnancy loss or neonatal death as well as lifelong hematologic, neurologic, dermatologic, and orthopedic sequelae in surviving infants without adequate treatment [2, 7]. Transmission is primarily transplacental in the setting of disseminated maternal infection rather than via exposure to syphilitic genital lesions at delivery [5]. Risk of CS increases with duration of gestational exposure and earlier stage of disease [5, 7]. Primary and secondary syphilis confer a 50%–70% risk of CS, compared with a 40% and 10% risk with early latent and late latent syphilis during pregnancy, respectively [7, 8].

In addition to prevention of syphilis with diagnosis and treatment before pregnancy, perinatal transmission of syphilis in pregnant people is preventable with early treatment using benzathine penicillin G, an evidence‐based, curative treatment for syphilis known to be safe in pregnancy and prevent CS by treating both the pregnant patient and the fetus [9, 10]. There is some evidence to suggest that cephalosporins such as ceftriaxone and cefixime cross the placenta and effectively treat pregnant patients with early‐stage syphilis, although further studies are needed [7, 11, 12]. However, benzathine penicillin G is the only treatment for syphilis in pregnancy that is currently recommended by the CDC [7]. The CDC also recommends empiric treatment for syphilis in certain populations, including those in areas or populations with high rates of syphilis or partners of individuals with syphilis of unknown duration and high nontreponemal titers (i.e., > 1:32). Additionally, persons who have had sexual contact with someone diagnosed with primary, secondary, or early latent syphilis in the 90 days preceding diagnosis should be empirically treated regardless of serology due to the risk for false‐negative results in the incubation period. Those with exposures more than 90 days prior should be empirically treated for syphilis if their serologies are unavailable or there is a high risk of loss to follow‐up [10].

Nearly 90% of all cases of CS in the United States in 2022 could have been prevented with timely screening and adequate treatment [2, 9, 13]. However, access to care is often complicated by barriers at both the individual level (e.g., housing instability, lack of transportation or childcare required to attend medical visits, or financial implications of missing work to seek medical treatment) and systems level (e.g., stigma surrounding sexually transmitted infections, inadequate health care coverage, and deep‐rooted mistrust in the medical system) [6, 9, 14]. Even with timely testing and accessible care, barriers such as complex diagnosis and treatment algorithms, difficulties accessing treatment history, unclear follow‐up requirements for subsequent testing of the pregnant person, and nationwide treatment shortages further impede treatment completion [9, 14]. For these reasons, ending the CS epidemic requires coordinated public health and clinical efforts.

## 2 WHY DIAGNOSIS AND TREATMENT ARE CHALLENGING

**FIGURE 1:**
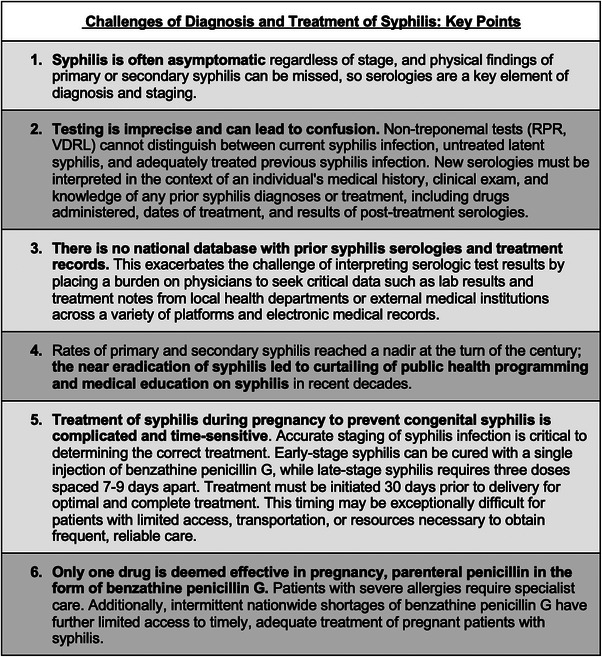
Key points to summarize the unique complexity of syphilis diagnosis and treatment.

The complexity of syphilis diagnosis poses a clinical and public health challenge, summarized in Figure 1. Presently, syphilis tests cannot distinguish between active infection, adequately treated previous syphilis infection, or untreated latent syphilis. As test results alone are inadequate to stage syphilis, and interpretations of testing may be even further complicated if tests are performed as rapid point‐of‐care tests, a clinician must interpret results in the context of each individual's history, clinical exam, and possibly past syphilis test results [14]. Variation in testing practices also exists, with some institutions using the traditional algorithm while others use the reverse algorithm to screen; both are acceptable according to the CDC, yet they have different advantages and disadvantages that vary by population [15]. This complexity contributes to clinical challenges with interpretation of serologic test results. Additionally, the lack of clinician access to a national database or reporting system for syphilis places a burden on clinicians to seek critical data such as lab results and treatment notes from local health departments or external medical institutions, which can be time consuming, confusing, or even impossible. Furthermore, the inherent challenges associated with the diagnosis and treatment of perinatal syphilis are compounded by many clinicians being unfamiliar with syphilis management. Despite a major public health response to rising rates of syphilis in the 1980s, declining rates of primary and secondary syphilis at the turn of the century led to curtailing of public health programming and medical education on syphilis in recent decades [5, 16]. This phenomenon is not new; in 1964, Dr. William J. Brown described this complex dilemma with regard to syphilis: “As a disease control program approaches the end point of eradication, it is the program, not the disease which is more likely to be eradicated….due to the disinterest of society in bearing that cost” [17].

Accurate diagnosis and staging of infection are imperative for adequate treatment. Although early‐stage syphilis can be cured with a single injection of benzathine penicillin G, late‐stage syphilis requires three doses, which in pregnancy must be spaced 7–9 days apart [2]. These rigid treatment intervals may be exceptionally difficult for patients with limited access, resources, or understanding necessary to adhere to treatment. Challenges with adherence are particularly problematic in pregnancy, given the limited time available to treat syphilis in the fetus before delivery; benzathine penicillin G must be initiated 30 days prior to delivery for optimal and complete treatment of the fetus. A 2023 article by McDonald et al. reported that out of 2179 pregnant patients who had timely identification of syphilis, more than two‐thirds had documentation of inadequate treatment due to inappropriate selection and dosing of antimicrobial agents, inaccurate timing of doses, or insufficient interval between treatment and delivery [2]. Together with intermittent nationwide shortages of benzathine penicillin G, these barriers amplify the disparity created by insufficient screening of pregnant patients in the United States and contribute to missed opportunities for prevention of CS [14, 18].

## 3 CURRENT SCREENING GUIDELINES AND LEGISLATION

Standardization of medical care based on best evidence can improve health outcomes, lower mortality, and promote equity. Nevertheless, there is a lack of standardized screening guidelines for syphilis during pregnancy in the United States.

Screening guidelines in the United States are variable across state lines, government agencies, and professional organizations. Current CDC guidelines recommend universal syphilis screening of pregnant patients in the first trimester (or earliest presentation to antenatal care) and repeat screening for high‐risk pregnant patients at 28 weeks’ gestation and at delivery [19]. “High risk” includes patients who live in communities with high rates of syphilis (> 4.6 per 100,000 people), have behavioral risk factors, or those not previously tested for syphilis during pregnancy [2, 19, 20]. Until recently, the American College of Obstetricians and Gynecologists (ACOG) advised universal early syphilis screening and repeat testing in select populations [4, 21]. In April 2024, ACOG shifted away from a risk‐based approach to screening by recommending universal re‐screening of pregnant people during the third trimester and at the time of delivery [9].

The ACOG update aligns with a growing body of evidence demonstrating clinicians are biased and inaccurate in their perception of who is at risk for infectious conditions such as syphilis, hepatitis C virus, and human immunodeficiency virus (HIV) [4, 6, 22, 23]. This inaccuracy is attributed to inadequate assessment of risk factors, clinician bias, and incomplete disclosure by patients [5, 24]. Behaviors used to classify patients as high risk (substance use, previous STI, new sexual partner, residence in high‐prevalence area, etc.) are intimately linked with social determinants of health such as socioeconomic status [6]. Yet in a review of 10,000 patients with antenatal syphilis, Trivedi et al. demonstrated that half reported having no high‐risk behaviors, highlighting the inadequacy of risk‐based assessment and need for universal screening [5, 6]. Universal screening may also decrease stigma associated with STI screening.

Other organizations, including the US Preventive Services Task Force (USPSTF), the Association of Women's Health, Obstetric and Neonatal Nurses (AWHONN), the American Academy of Family Physicians (AAFP), and the American Academy of Pediatrics (AAP), also have guidelines for the screening and management of syphilis in pregnancy. The USPSTF released an updated grade‐A recommendation in May of 2025, which reaffirmed its 2018 grade‐A recommendation for early, universal screening of pregnant patients for syphilis [25]. This recommendation was endorsed by the AAFP [26, 27]. In 2023, AWHONN published an open letter urging health care providers to emphasize the importance of universal first‐trimester screening for syphilis with additional screening of high‐risk populations later in pregnancy [28]. The AAP, in conjunction with ACOG in 2017, similarly recommends universal first‐trimester screening and repeat screening for high‐risk populations [21].

**FIGURE 2:**
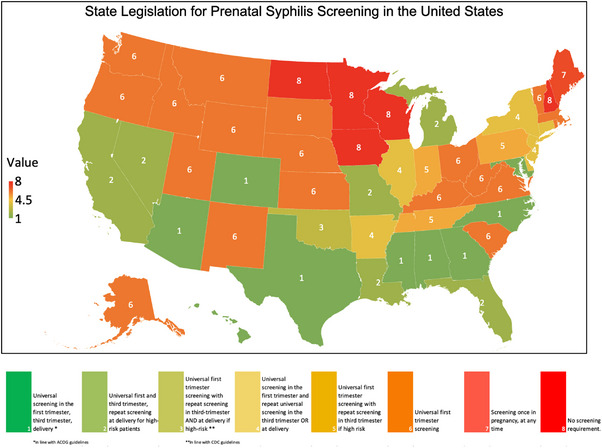
Existing legislation for prenatal syphilis screening in the United States. The degree of existing legislation increases on this scale from green to red. Scale is determined by numerical grading from 1 (dark green) to 8 (dark red), as defined in the key.

Mandating screening through legislation has effectively improved public health practice for some conditions [22, 29, 30, 31]. A review of current US legislation (50 states and Washington, DC) for syphilis screening during pregnancy is shown in Table 1. Less than half (46%) of states’ legislation meets CDC guidelines and, acknowledging that the current ACOG guidelines are new, only 17% (9) of states have legislation aligned with the 2024 ACOG recommendations for universal screening at three time points (first visit, third trimester, and delivery) [1]. Forty‐five states require universal testing at the initial prenatal visit; of those, 21 mandate additional universal screening in the third trimester, and 10 require testing at delivery [1, 32]. Maine requires testing at least once in pregnancy but does not specify timing [1]. Five states (IA, MN, NH, ND, and WI) have no syphilis screening requirement during pregnancy. Ten states use a risk‐based approach to re‐screening after the initial visit [1]. Legislation for prenatal syphilis screening in each state is demonstrated in Figure 2.

**TABLE 1:** Legal requirements for serologic testing for syphilis in pregnancy among the 50 states and Washington, DC, adapted from the 2020 CDC report titled “State Statutory and Regulatory Language Regarding Prenatal Syphilis Screenings in the United States,” which was last updated in June 2025 [1].

State	First visit	Third trimester	Delivery
Alabama	X	X	X
Alaska	X		
Arizona	X	X	X
Arkansas	X	X	
California	X	X	O
Colorado	X	X	X
Connecticut	X	X	
Delaware	X	X	
DC	X	X	
Florida	X	X	O
Georgia	X	X	X
Hawaii	X	X	X
Idaho	X		
Illinois	X	X	
Indiana	X	O	
Iowa			
Kansas	X		
Kentucky	X		
Louisiana	X	X	O
Maine	*	*	*
Maryland	X	X	X
Massachusetts	X		
Michigan	X	X	O
Minnesota			
Mississippi	X	X	X
Missouri	X	X	O
Montana	X		
Nebraska	X		
Nevada	X	X	O
New Hampshire			
New Jersey	X		X
New Mexico	X		
New York	X	X	
North Carolina	X	X	X
North Dakota			
Ohio	X		
Oklahoma	X	O	O
Oregon	X		
Pennsylvania	X	O	
Rhode Island	X		
South Carolina	X		
South Dakota	X		
Tennessee	X	O	
Texas	X	X	X
Utah	X		
Vermont	X		
Virginia	X		
Washington	X		
West Virginia	X		
Wisconsin			
Wyoming	X		

*Note*: X denotes required universal screening, O denotes screening for high‐risk patients only, and * denotes screening once in pregnancy at an unspecified time. Empty cell designates no legislation regarding syphilis screening at that time point.

**FIGURE 3:**
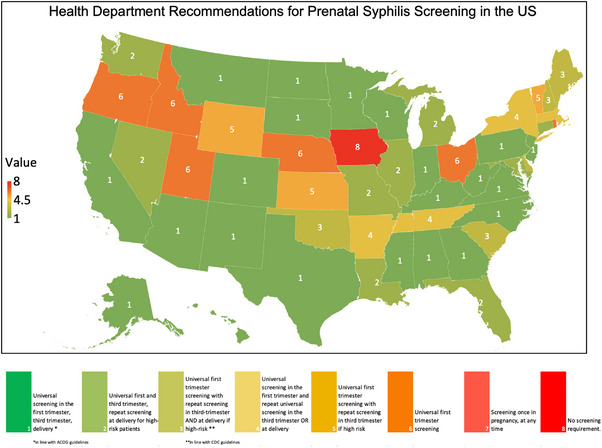
Public health advisory recommendations for prenatal syphilis screenings in the United States. The degree of existing legislation increases on this scale from green to red. Scale is determined by numerical grading from 1 (dark green) to 8 (dark red), as defined in the key.

In response to rising CS rates between 2019 and 2024, health departments in 25 states issued alerts or advisories recommending more frequent prenatal syphilis screening than is required by their states’ existing legislation, shown in Figure 3 [33, 34, 35, 36, 37, 38, 39, 40, 41, 42, 43, 44, 45, 46, 47, 48, 49, 50, 51, 52, 53, 54, 55, 56, 57, 58]. Among those are four of the five states with no legislation requiring antenatal screening; three (MN, ND, and WI) now encourage universal screening in accordance with ACOG's new guidelines. When combining health alerts/advisories with legislation, the percentage of states whose guidelines align with CDC guidelines increases from 46% to 80%; similarly, when considering health alerts/advisories, the percentage of states whose guidelines align with ACOG rises from 17% to 43%. This contrast, shown clearly by comparing Figures 2 and 3, exemplifies the way US legislation frequently lags behind public health practice.

## 4 IS LEGISLATION ENOUGH?

Although opt‐out prenatal screening and legislation are important in combating the syphilis epidemic, they do not provide equitable benefit to all patients. Differences in rates of syphilis based on race and ethnicity, for example, reflect complex underlying causes including disparities in access to health care [2]. The missed opportunities contributing to the rise in CS in the United States today are most commonly due to delayed or a lack of prenatal care. Such gaps include prenatal care without screening as well as late identification of syphilis and inadequate (incomplete and/or mistimed) treatment during pregnancy [5, 14]. Consequently, legislation mandating antenatal syphilis screening without additional resources to support clinicians, reduce community transmission, and improve patients’ access to care is insufficient to combat rising rates of syphilis and CS. In addition, some jurisdictions may not have legislation for syphilis testing because they consider testing for syphilis to be the realm of public health departments and not appropriate for legislation.

The need for additional resources and public health support to prevent perinatal transmission of syphilis was demonstrated in a retrospective cohort study by Clement et al., which found that, despite a decades‐old mandate in Illinois for universal third‐trimester syphilis screening in a high‐prevalence region, adherence to screening guidelines was suboptimal and biased [4]. Among patients who delivered at a large‐volume, urban academic medical center over the three‐year study period, nearly 70% fewer participants had screening in the third trimester compared to the first trimester (96.9% first trimester and 27.3% third trimester). Additionally, significant demographic differences existed between those who did and did not receive third‐trimester screening, where younger, non‐White, non‐English speaking, publicly insured, multiparous women were more likely to have repeat screening in the third trimester. These findings highlight the role that clinician bias plays in compliance with state‐mandated testing and raise questions about the adequacy of universal screening legislation alone as a means to create equitable, high‐quality care for all pregnant people and their infants [4, 22, 23].

## 5 CONCLUSIONS AND FUTURE DIRECTIONS

In this article, we reviewed the CS crisis and corresponding legislation across the country, highlighting gaps and between‐state differences in legislation and resources available to clinicians who diagnose and treat syphilis in the perinatal period. However, the fight against the current CS crisis will require more than inclusive and uniform legislation. It will also be imperative to address the social needs of patients and communities and provide the resources necessary to adequately test and treat community members in a timely manner.

Field‐delivered therapy is one promising strategy to expand access and increase the likelihood of adequate treatment for individuals in rural areas or for those with barriers to completing treatment at the rigid intervals required to treat late latent syphilis [2, 13, 16]. Currently, field‐delivered therapy is infrequently used for syphilis for a variety of reasons, including the need for injections and the low but present risk for the Jarisch‐Herxheimer reaction—an acute inflammatory reaction to cytokines and lipoproteins released after initiation of antibiotic treatment causes widespread lysis of spirochetes in the body [59, 60, 61, 62, 63]. However, field‐delivered therapy may be a promising approach for the expansion of treatment with clinical precautions and appropriate expertise. Many of the authors of the present report have contributed to the ongoing development of a field treatment program in Illinois aimed at facilitating at‐home administration of penicillin to pregnant patients. Additional opportunities for public health programming to combat rising rates of syphilis and CS may include point‐of‐care testing for partners and/or expedited partner therapy (EPT), which provides treatment to sex partners without a visit to a health care provider. EPT is not currently approved for use with syphilis as its treatment usually requires an injection; however, this may change with new partner testing and treatment initiatives.

The state of Illinois provides an example of an effective and achievable robust public health approach to this crisis. Through the ongoing efforts of a statewide action team, Illinois is developing initiatives (e.g., field‐delivered therapy, point‐of‐care‐testing, EPT, resource navigation, centralized access to patient histories and reporting) aimed at tackling barriers to care and treatment, while also addressing the need for expert clinical support. Illinois has utilized the infrastructure of an existing state‐funded clinical consultation hotline for perinatal HIV prevention as a blueprint for the development of a similar program for perinatal syphilis: the Perinatal Syphilis Warmline [64, 65]. Launched in November 2023, the Warmline is led by a public health and academic partnership involving maternal‐fetal medicine, adult infectious disease, and pediatric infectious disease experts. It guides adult and pediatric clinicians through the complex landscape of perinatal syphilis diagnosis, staging, treatment, and reporting. This program demonstrates a public health and clinical partnership providing complex and time‐sensitive clinical care for pregnant people. A similar program is the STD Clinical Consultation Network, which is a national consultation service to provide guidance to clinicians [66]. Although both the Warmline and the STD Clinical Consultation Network help address the need for expanded access to expert care, the Warmline is able to provide Illinois clinicians with access to patients’ prior syphilis testing and treatment records through its partnership with public health authorities. Leaders of the Illinois Perinatal Syphilis Warmline hope to study its impacts over time to document the program's effect and identify areas for quality improvement.

It is our hope that updated legislation, public health advisories, and clinical practice guidelines combined with strengthened nationwide public health programming for CS prevention – through both local and state initiatives – will provide the necessary support to patients and health care providers to combat the crisis and prevent the devastating health impacts of syphilis during pregnancy and CS.

## CONFLICT OF INTEREST STATEMENT

The authors declare no conflicts of interest.
